# Chromosome studies in Orchidaceae from Argentina

**DOI:** 10.1590/S1415-47572009005000089

**Published:** 2009-12-01

**Authors:** Julio Rubén Daviña, Mauro Grabiele, Juan Carlos Cerutti, Diego Hernán Hojsgaard, Rubén Dario Almada, Irma Stella Insaurralde, Ana Isabel Honfi

**Affiliations:** 1 Programa de Estudios Florísticos y Genética Vegetal, Universidad Nacional de Misiones, PosadasArgentina; 2 Instituto Multidisciplinario de Biología Vegetal, Universidad Nacional de Córdoba, CórdobaArgentina; 3 Instituto de Botánica del Nordeste, Universidad Nacional del Nordeste, CorrientesArgentina; 4 Instituto de Biología Vegetal y Biotecnología, Universidad de Talca, TalcaChile

**Keywords:** chromosome number, karyotype features, polyploidy, karyology, orchids, South America

## Abstract

The center of diversity of Argentinean orchids is in the northeast region of the country. Chromosome numbers and karyotype features of 43 species belonging to 28 genera are presented here. Five chromosome records are the first ones at the genus level; these taxa are *Aspidogyne kuckzinskii* (2*n* = 42), *Eurystyles actinosophila* (2*n* = 56), *Skeptrostachys paraguayensis* (2*n* = 46), *Stigmatosema polyaden* (2*n* = 40) and *Zygostates alleniana* (2*n* = 54). In addition, a chromosome number is presented for the first time for 15 species: *Corymborkis flava* (2*n* = 56), *Cyclopogon callophyllus* (2*n* = 28), *C. oliganthus* (2*n* = 64), *Cyrtopodium hatschbachii* (2*n* = 46), *C. palmifrons* (2*n* = 46), *Galeandra beyrichii* (2*n* = 54), *Habenaria bractescens* (2*n* = 44), *Oncidium edwallii* (2*n* = 42), *O. fimbriatum* (2*n* = 56), *O. pubes* (2*n* = 84), *O. riograndense* (2*n* = 56), *Pelexia ekmanii* (2*n* = 46), *P. lindmanii* (2*n* = 46) and *Warrea warreana* (2*n* = 48). For *Oncidium longicornu* (2*n* = 42)*, O. divaricatum* (2*n* = 56) and *Sarcoglottis fasciculata* (2*n* = 46+1B?, 46+3B?), a new cytotype was found. Chromosome data support phylogenetic relationships proposed by previous cytological, morphologic and molecular analyses, and in all the cases cover some gaps in the South American literature on orchid chromosomes.

## Introduction

Orchidaceae Juss. is one of the largest families in the plant kingdom, distributed throughout the tropical and subtropical areas of both hemispheres (Correa, 1955). The family comprises around 850 genera and 20000 species, and nearly 75% of them are epiphytes (Dressler, 1993; Johnson, 2001). In Argentina, this family comprises around 74 genera and *c.* 280 species (Johnson, 1992; Correa, 1996) distributed throughout the country. Numerous orchids are part of the northeast Argentinean flora, particularly that of the provinces of Misiones (154 species), Corrientes (76), and Chaco (34) (Correa, 1996; Zuloaga *et al.*, 1999; Insaurralde and Radins, 2007), and most of them have ornamental value.

Cytogenetic studies on Orchidaceae are few, disperse and incomplete. It is estimated that so far no more than about 10% of all species were chromosomally analyzed, therefore the chromosome evolution in this family remains in debate. The chromosome numbers vary from 2*n* = 10 to 2*n* = 240 and high numbers are common, the most frequent being *n* = 19 or 20 (Jones, 1974). Regarding chromosome studies of South American orchids, the most important contributions were made by Blumenschein (Blumenschein A, PhD Thesis, University of São Paulo, Piracicaba, 1957; Blumenschein, 1960), Martínez (1981, 1985), Dematteis and Daviña (1999) and Felix and Guerra (1998, 1999, 2000, 2005), but the data are still considered insufficient. The cytology of orchids has been considered in the past to have a great potential in studies of taxonomic affinities and evolutionary bonds (Jones, 1974), and this is still true today, mainly if we take into account the phylogenetic gaps or misinterpretations of evolutionary trends. Moreover, in northeast Argentina, a great number of orchid populations are currently limited to remnants of the subtropical forest. Both epiphytes and terrestrial species grow in vegetation fragments, and their conservation is difficult due to changes in the habitat, patchy distribution and specific pollination strategies. For these reasons and as a first step towards characterization of the genetic variability of the species belonging to South American orchid genera, a cytological study was carried out.

## Material and Methods

Forty-three species of Orchidaceae from Argentina were chromosomally studied (Table 1). Samples were cultivated in the greenhouse of the Programa de Estudios Floristicos y Genetica Vegetal (FCEQyN - UNaM), and some of them have not flowered yet. Voucher specimens were deposited at the herbarium of the Universidad Nacional de Misiones (MNES). The taxonomic nomenclature adopted for the species was that of Govaerts *et al.* (2008). The results obtained are presented and discussed according to the classifications of Dressler (1993) and/or Szlachetko (1995) at suprageneric levels. Mitotic studies were performed in root tips pretreated with saturated 1-bromonaphthalene for 2-3 hs at room temperature, fixed in absolute ethanol:glacial acetic acid (3:1) for 12 hs at 4 °C, and stained according to the Feulgen technique. The meristems were macerated in a drop of 2% aceto-orcein and then squashed. Permanent slides were made using euparal as a mounting medium. Slides were analyzed with a Leica DMLS optical photomicroscope, photographs were taken with Imagelink HQ 25 ASA Kodak film, and negatives were digitalized with a Genius ColorPage-HR8 scanner. Chromosome measurements were made using the MicroMeasure 3.3 computer program (Reeves, 2001).

## Results

**Figure 1:**
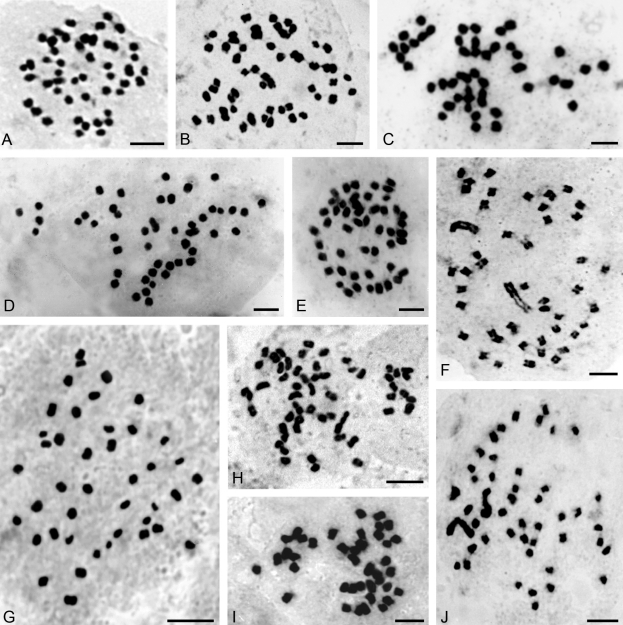
Somatic chromosomes of: (A) *Pelexia lindmanii* (2*n* = 46), (B) *P. ekmanii* (2*n* = 46), (C) *Sarcoglottis fasciculata* (2*n* = 46), (D) *S. fasciculata* (2*n* = 47), (E) *S. fasciculata* (2*n* = 49), (F) *Mesadenella cuspidata* (2*n* = 46), (G) *Aspidogyne kuczynskii* (2*n* = 42), (H) *Eurystyles actinosophila* (2*n* = 56), (I) *Stigmatosema polyaden* (2*n* = 40), and (J) *Skeptrostachys paraguayensis* (2*n* = 46). Scale bars = 5 μm.

The 43 species of orchids chromosomally studied in this work belong to 28 genera distributed into five subfamilies: Epidendroideae (three species: *Brassavola, Leptotes, Sophronitis*), Orchidoideae (one; *Habenaria*), Spiranthoideae (17: *Aspidogyne, Cyclopogon, Eltroplectris, Eurystyles, Mesadenella, Pelexia, Sacoila, Sarcoglottis, Skeptrostachys, Stigmatosema*), Tropidoideae (one; *Corymborkis*) and Vandoideae (21: *Campylocentrum, Catasetum, Cyrtopodium, Galeandra, Gomesa, Oeceoclades, Oncidium, Miltonia, Rodriguezia, Trichocentrum, Warrea, Zygopetalum, Zygostates*) and are summarized in Table 1. Selected photomicrographs of the chromosomes of some species are shown in Figures 1 and 3.

**Figure 2:**
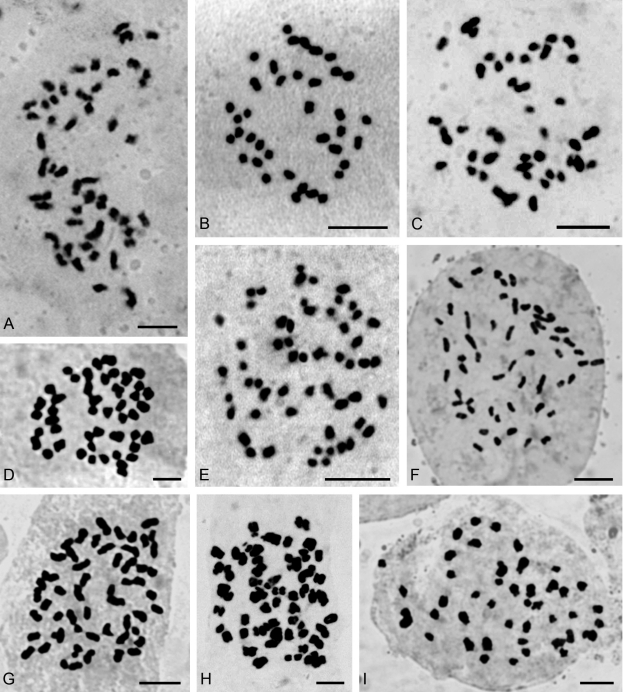
Somatic chromosomes of: (A) *Warrea warreana* (2*n* = 48), (B) *Campylocentrum neglectum* (2*n* = 38), (C) *Rodriguezia decora* (2*n* = 42), (D) *Cyrtopodium hatschbachii* (2*n* = 46), (E), *Oeceoclades maculata* (2*n* = 56), (F) *Zygostates alleniana* (2*n* = 54), (G) *Galeandra beyrichii* (2*n* = 54), (H), *Corymborkis flava* (2*n* = 56), and (I) *Habenaria bractescens* (2*n* = 44). Scale bars = 5 μm.

**Figure 3:**
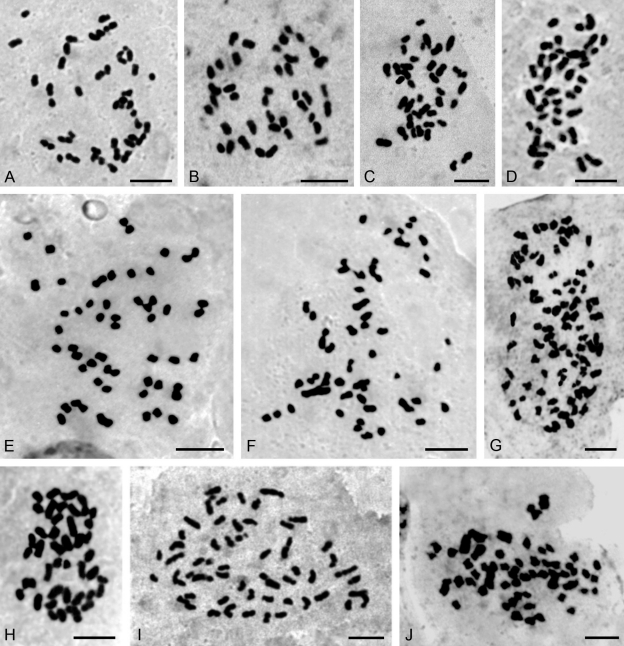
Somatic chromosomes of: (A) *Brassavola tuberculata* (2*n* = 40), (B) *Leptotes unicolor* (2*n* = 40), (C), *Sophronitis cernua* (2*n* = 40), (D) *Oncidium longicornu* (2*n* = 42), (E) *O. fimbriatum* (2*n* = 56), (F) *O. divaricatum* (2*n* = 56), (G) *O. pubes* (2*n* = 84), (H) *O. edwalli* (2*n* = 42), (I) *Gomesa planifolia* (2*n* = 56), and (J) *Miltonia flavescens* (2*n* = 60). Scale bars = 5 μm.

Five chromosome counts are first records at genus level; these species are: *Aspidogyne kuckzinskii* (2*n* = 42) (Figure 1G), *Eurystyles actinosophila* (2*n* = 56) (Figure 1H), *Skeptrostachys paraguayensis* (2*n* = 46) (Figure 1J), *Stigmatosema polyaden* (2*n* = 40) (Figure 1I) and *Zygostates alleniana* (2*n* = 54) (Figure 2F). Fifteen additional species had not yet been studied chromosomally, so this is the first record too: *Corymborkis flava* (2*n* = 56) (Figure 2H), *Cyclopogon callophyllus* (2*n* = 28), *C. oliganthus* (2*n* = 64), *Cyrtopodium hatschbachii* (2*n* = 46) (Figure 2D), *C. palmifrons* (2*n* = 46), *Galeandra beyrichii* (2*n* = 54) (Figure 2G), *Gomesa planifolia* (2*n* = 56) (Figure 3I), *Habenaria bractescens* (2*n* = 44) (Figure 2I), *Oncidium fimbriatum* (2*n* = 56) (Figure 3E), *O. pubes* (2*n* = 84) (Figure 3G), *O. edwallii* (2*n* = 42) (Figure 3H), *O. riograndense* (2*n* = 56), *Pelexia lindmanii* (2*n* = 46) (Figure 1A), *P. ekmanii* (2*n* = 46) (Figure 1B), and *Warrea warreana* (2*n* = 48) (Figure 2A). For *Oncidium longicornu* (2*n* = 42) (Figure 3D), *O. divaricatum* (2*n* = 56) (Figure 3F) and *Sarcoglottis fasciculata* (2*n* = 46, 46+1B?, 46+3B?), new cytotypes were found (Figure 1C,D,E). Many results (20) are in accordance with previous chromosome reports, but some of them (7) are first records for Argentinean orchid populations, like *Brassavola tuberculata* (2*n* = 40) (Figure 3A), *Campylocentrum neglectum* (2*n* = 38) (Figure 2B), *Leptotes unicolor* (2*n* = 40) (Figure 3B), *Miltonia flavescens* (2*n* = 60) (Figure 3J), *Rodriguezia decora* (2*n* = 42) (Figure 2C), *Sophronitis cernua* (2*n* = 40) (Figure 3C), and *Zygopetalum maxillare* (2*n* = 48). Furthermore, we also found a 2*n* = 56 cytotype in *Oeceoclades maculata* populations, which differs from previous counts performed in Brazilian materials (Figure 2E).

In most of the analized taxa, the karyotype was found to be of the bimodal type. Bimodality is present to varying degrees, as evidenced by the short/large chromosome ratio (S/L). Some Epidendroideae species showed small chromosomes, ranging from 0.5 to 2.5 μm (S/L = 0.20). The chromosomes of *Habenaria* (Orchidoideae) were small, ranging from 1 to 3 μm (S/L = 0.33), whereas those of *Corymborkis* (Tropidoideae) were of median size, ranging from 1 to 4 μm (S/L = 0.25). In Vandoideae, most of the species showed small chromosomes, ranging from 0.5 to 2.5 μm (S/L = 0.20), except for *Gomesa* (small to median, 0.5 to 4 μm; S/L = 0.13) and *Warrea* and *Zygopetalum* (median size, 0.5 to 3 μm; S/L = 0.17). The Spiranthoideae species showed the widest variation regarding both chromosome size and interchromosomal asymmetry. *Cyclopogon* has median-size chromosomes (1.75 to 3.5 μm; S/L = 0.50), just as *Pelexia* and *Sarcoglottis* (1.4 to 3.7 μm; S/L = 0.38), whereas *Eltroplectris schlechteriana* (2*n* = 26), *Mesadenella cuspidata* (2*n* = 46, Figure 1F), *Sacoilalanceolata* and *Skeptrostachysparaguayensis* present small to large chromosomes (1.25 to 5 μm; S/L = 0.25), with one chromosome pair that is twice as large as the chromosome mean and carries a macrosatellite on the short arm. The other genera exhibit small chromosomes, as follows: *Aspidogyne* (0.5 to 2 μm; S/L = 0.25), *Eurystyles* (0.5 to 2.5 μm; S/L = 0.20) and *Stigmatosema* (1 to 2.5 μm; S/L = 0.40).

## Discussion

A wide diversity in chromosome numbers and karyotype features was found among the species of orchids that inhabit the northeast region of Argentina. In order to clarify any possible relationships among taxa, the results obtained are discussed, first circumscribed to each subfamily as starting point and then as a whole.

Epidendroideae is a large subfamily that comprises about 220 genera and the most common chromosome numbers are 2*n* = 28, 30, 32, 34, 36, 38, 40, 42, 46, 48, 50 (Szlachetko, 1995). From this clade, we analyzed three species, *Brassavola tuberculata*, *Leptotes unicolor* and *Sophronitis cernua,* and our chromosome count (2*n* = 40) agrees with previous reports (Afzelius, 1943; Blumenschein, 1960). These three genera belong to the subtribe Laeliinae (tribe Epidendreae) that comprises around 31 genera and 1466 species (Dressler, 1993; Szlachetko, 1995). The chromosome number in the neotropical orchid subtribe Laeliinae varies from 2*n* = 24 to 2*n* = 56, but is most commonly 2*n* = 40 (Tanaka and Kamemoto, 1984). Our chromosome records, along with previous ones obtained for these genera (Afzelius, 1943; Kamemoto, 1950; Mehlquist, 1953; Blumenschein, 1960; Chardard, 1963), support the hypothesis that 2*n* = 40 is the most common number for Laeliinae (Cameron *et al.*, 1999; Dematteis and Daviña, 1999), for which the basic chromosome number could be a multiple of *x* = 10.

The Orchidoideae subfamily cromprises around 78 genera, including *Habenaria*, and the most common chromosome numbers are 2*n* = 30, 32, 36, 38, 40, 42, 44, 46, 48 (Szlachetko, 1995). *Habenaria* (tribe Orchideae) is a cosmopolitan genus with around 600-800 terrestrial species, of which 21 occur in Argentina (Correa, 1996; Felix and Guerra, 1998; Johnson, 2001). The chromosome count found for *H.bractescens* (2*n* = 44), which is the first one for this species, is present in only 7% of the chromosomally analyzed *Habenaria* species, as this genus shows a wide diversity of cytotypes (35), ranging from 2*n* = 28 (Tanaka, 1971) to 168 (Mehra and Sehgal, 1974), and the most common are *n* = 21 and 2*n* = 42 (Felix and Guerra, 1998).

Subfamily Spiranthoideae encompasses about 95 genera and 1140 species, predominantly terrestrial (Salazar *et al.*, 2003), with 2*n* = 24, 26, 28, 30, 32, 36, 44, and 46 as the most common chromosome numbers (Szlachetko, 1995). However, Cameron *et al.* (1999) did not recognize this clade as a separate subfamily and included it within Orchidoideae. We analyzed 17 species of 10 genera and found a wide diversity of chromosome numbers, *i.e.*, 2*n* = 26, 28, 32, 40, 42, 46, 56, 64. This is the first time chromosome counts of *Aspidogyne, Eurystyles* and *Stigmatosema* species were made, but more studies are needed on these neglected genera. In natural populations of *Aspidogynekuczynskii*, we observed polymorphism for the color of the leaves among different plants, but all phenotypes presented the same chromosome number.

The type of karyotype bimodality observed in *Sacoila,Skeptrostachys, Mesadenella* and *Eltroplectris,* with a chromosome pair twice as large as the mean chromosome size and carrying a macrosatellite on the short arm, along with previous cytogenetical data (Cocucci, 1956; Martínez, 1985; Dematteis and Daviña, 1999; Felix and Guerra, 2005), support their inclusion in a separate clade (subtribe Stenorrynchidinae *sensu* Szlachetko, 1995; Stenorrhynchos clade *sensu* Salazar *et al.*, 2003). On the other hand, *Pelexia bonariensis*, *P. ekmanii*, *P. lindmanii*, *Sarcoglottis fasciculata*, *S. grandiflora* and *S. ventricosa* also share the chromosome number (2*n* = 46) and the general karyotype features, which, together with previous cytogenetic data for those genera (Martínez, 1985; Dematteis and Daviña, 1999; Felix and Guerra, 2005), reinforces the proposal incorporating *Pelexia* and *Sarcoglottis* into the same clade (Dressler, 1993; Szlachetko, 1995; Salazar *et al.*, 2003). Moreover, in *S. fasciculata* we found 2*n* = 46, 47 and 49 in the same plant. This variation in the somatic chromosome number may be due to the presence of B chromosomes with mitotic instability, as reported in several angiosperms (Jones and Rees, 1982), or aneusomaty.

The four *Cyclopogon* species analyzed here showed 2*n* = 28 (*C. callophyllus,* and *C. elatus*), 32 (*C. congestus*) and 64 (*C. oliganthus*), representing new records for the first and the latter species. *Cyclopogon* is a taxonomically complex genus, as evidenced by morphological and molecular data (Dressler, 1993; Szlachetko, 1995; Salazar *et al.*, 2003). In addition, it displays a wide diversity of chromosome numbers and ploidy levels, *n* = 14, 16 and 2*n* = 28, 30, 32, 36, 45, 64 (Martínez, 1981, 1985; Tanaka and Maekawa, 1983; Felix and Guerra, 2005; this work). For these reasons, detailed cytogenetical analyses to circumscribe this taxon are needed.

Tropidoideae is a small subfamily that comprises three genera including *Corymborkis* (tribe Tropideae) (Szlachetko, 1995). Cameron *et al.* (1999), however, did not support this clade as a separate subfamily, but as a tribe of Epidendroideae. The chromosome count for *Corymborkis flava* (2*n* = 56) reported here is the first one for this species. Only other two chromosome counts were made in this genus (2*n* = 40, 58), for *C.veratrifolia*, by Pancho (1965) and Ono and Masuda (1981), respectively.

The Vandoideae subfamily comprises around 347 genera distributed into 12 tribes, forming a monophyletic group with 2*n* = 38, 40, 42, 46, 48, 50, 52, 54, 56 as the most common chromosome numbers (Szlachetko, 1995). Again, Cameron *et al.* (1999) did not recognize this clade as a separate subfamily and included it within Epidendroideae. We analyzed 21 species of 13 genera and found a wide diversity of chromosome numbers ranging from 2*n* = 30 to 108.

Among other genera, the tribe Cymbidieae encompasses *Cyrtopodium*, *Galeandra* and *Oeceoclades* (Dressler, 1993; Szlachetko, 1995). For *Cyrtopodium*, only 10 species had their chromosomes previously counted, showing *n* = 22 and 2*n* = 46, 92 (Aoyama, 1989; Felix and Guerra, 2000). *Cyrtopodium hatschbachii* and *C. palmifrons* presented 2*n* = 46, which, combined with the 23II observed at diakinesis in the former species (unpublished data), allows us to propose a basic chromosome number of *x* = 23 for this genus, although it might be a derived number. For *Galeandra*, there are only two previous chromosome reports concerning *G. baueri* and *G. devoniana,* two epiphytic species with 2*n* = 56 (Aoyama, 1989). Our count (2*n* = 54) in *G. beyrichii,* a terrestrial species, suggests that there is probably more than one basic chromosome number in this genus. As in *Galeandra*, Felix and Guerra (2000) report different chromosome numbers for Brazilian *Cyrtopodium* and *Oncidium* orchids with different habitats. Genus *Oeceoclades* comprises about 39 species distributed over Africa, and *O. maculata* is the only one that also lives in America (Govaerts *et al.*, 2008). In Brazilian *O. maculata* populations, 2*n* = 48, c.52, 54, 58 cytotypes were found (Guerra, 1986; Felix and Guerra, 2000). However, for Argentinean populations, we consistently observed a 2*n* = 56 cytotype, in agreement with our previous work (Dematteis and Daviña, 1999). Our results regarding *O. maculata* agree with the proposal of the primary basic number *x*_*1*_ = 7 for Cymbidieae, presented by Felix and Guerra (2000), so this species could be considered an octoploid.

*Catasetum* (tribe Catasetinae) is an American genus with 163 species (Govaerts *et al.*, 2008), and 2*n* = 54 is the most common chromosome number. In *C. fimbriatum*, we observed 2*n* = 108. The available chromosome data for the genus (Jones and Darker, 1968; Dematteis and Daviña, 1999; Felix and Guerra, 2000) suggest a polyploid series, probably based on x = 6. Cameron *et al.* (1999) and Cameron (2004) support the idea that genus *Galeandra* (Cymbidieae) is closer to *Catasetum* than to *Cyrtopodium* (Cymbidieae), and our chromosome counts reinforce their proposal.

*Warrea warreana* and *Zygopetalum maxillare* presented 2*n* = 48 median-size chromosomes. Both genera are included in the same clade, tribe Maxillarieae, according to Dressler (1993), or Zygopetaleae *sensu* Szlachetko (1995). Our cytogenetic data, along with previous chromosome number reports for both genera (Blumenschein, 1960; Tanaka and Kamemoto, 1984; Aoyama, 1989; Aoyama *et al.*, 1994), support the idea of their inclusion in the same clade and reinforce the hypothesis of *x* = 24 or 26 in this group (Felix and Guerra, 2000).

*Campylocentrum neglectum* (tribe Vandeae) showed a 2*n* = 38, and this is the first count for natural populations from Argentina. The chromosome number agrees with that from Paraguay reported by Dematteis and Daviña (1999). No other *Campylocentrum* species were chromosomally analyzed so far, although 64 tropical and subtropical American species of this genus were described.

*Zygostates alleniana* (tribe Maxillarieae *sensu* Dressler, 1993; tribe Ornithocephaleae *sensu* Szlachetko, 1995) presented 2*n* = 54 small chromosomes, and this is the first count for the genus.

The subtribe Oncidiinae is taxonomically complex (Dressler, 1993; Szlachetko, 1995; Felix and Guerra, 2000; Chase *et al.*, 2005). Both Dressler (1993) and Szlachetko (1995) recognize this clade, but the former included it within the tribe Maxillarieae of the Cymbidioid phylad, and the latter within the Vandoideae tribe Oncidieae. The variability in chromosome numbers is a representative feature of Oncidiinae, showing the widest range of variation within orchids, from 2*n* = 10 to 168, but 2*n* = 56, 60 and 42 are the most common within a polyploid series based on *x* = 7 (Felix and Guerra, 2000). We analyzed species of *Gomesa*, *Miltonia*, *Oncidium*, *Rodriguezia* and *Trichocentrum* (subtribe Oncidiinae *sensu* Dressler, 1993; tribe Oncidieae *sensu* Szlachetko, 1995) and found 2*n* = 30, 42, 60, 84, 108, emphasizing the proposal of *x* = 7 for this clade made by Felix and Guerra (2000).

*Oncidium* is a large neotropical genus with showy epiphytic, litophytic and terrestrial species, and some of them can be misidentified when in vegetative state. This genus has been extensively studied, showing 2*n* = 56, 42, 28 as the most common chromosome numbers (Felix and Guerra, 2000). All the *Oncidium* species analyzed here showed small chromosome size (0.5 to 3 μm) and presented 2*n* = 42, 56 as the most common numbers, with the exception of *O. bifolium* (2*n* = 108) and *O. pubes* (2*n* = 84). Cytotype 2*n* = 84 is present in at least six species (Tanaka and Kamemoto, 1984; Felix and Guerra, 2000) that could be considered 12-ploid, whereas cytotype 2*n* = 108 could be considered a 16-ploid derived from 2*n* = 112, both based on *x* = 7.

*Trichocentrum pumilum* showed 2*n* = 30, which is in agreement with previous records (as *Oncidium pumilum*, Dematteis, 1997; Dematteis and Daviña, 1999; Felix and Guerra, 2000); cytological data sustain the exclusion of *O. pumilum* from genus *Oncidium*, in accordance with molecular data (Williams *et al.*, 2001).

*Rodrigueziadecora* was found to have 2*n* = 42, and this is the first count for natural populations from Argentina. Sinotô (1962) previously reported the same number for Brazilian populations. Only eight species of *Rodriguezia* had their chromosomes counted, consistently showing 2*n* = 42 (Felix and Guerra, 2000). The chromosome count 2*n* = 56 is the first one for *Gomesa planifolia*. The chromosomes of this genus were nearly unknown, since only two other species were previously counted: *G. recurva* and *G. crispa* (2*n* = 56; Sinotô, 1962, 1969; Charanasri *et al.*, 1973; Tanaka and Kamemoto, 1984). *Miltonia flavescens* showed 2*n* = 60, and this is the first count for natural populations from Argentina, coinciding with that of Sinotô (1962, 1969), Charanasri *et al.* (1973) and Félix and Guerra (2000). Eight *Miltonia* species had their chromosome counts reported, with 2*n* = 60 and 2*n* = 56, in that order, as the most common numbers, but 2*n* = 59, 86 were also found (Sinotô, 1962, 1969; Charanasri *et al.*, 1973; Charanasri and Kamemoto, 1975). Felix and Guerra (2000) proposed that the *Miltonia* species which had their chromosomes counted are octoploids from *x* = 7, and dysploidy is thought to explain the cytotype 2*n* = 60.

At any stage of analysis, Orchidaceae is a complex taxon. Its large number of species, along with a wide variation at the morphological, ecological and cytological levels, have made it difficult to establish an accurate phylogenetic scenario using classical approaches. Therefore, different criteria for the classification of these orchids as a whole have been applied, such as those of Dressler (1993) and Szlachetko (1995) as the most recent analyses, resulting in different evolutionary pictures. Molecular analysis of the orchids as a whole (*i.e.*, Cameron *et al.*, 1999; Cameron, 2004; Chase *et al.*,2005) or of any of their larger clades (*i.e.*, Cameron and Chase, 2000; Chase *et al.*, 2005; Van den Berg *et al.*, 2005) proved to aid the classical approaches, but differing at some point. Regarding the number of subfamilies comprised by Orchidaceae, several contradictions have arisen between classical and molecular analysis. Dressler (1993) and Szlachetko (1995) do not assign the genera to the same taxonomic intra-family categories, whereas Cameron *et al.* (1999) support five major monophyletic clades, delimiting some taxa sustained by the former authors (such as inclusion of Vandoideae and Tropidoideae into Epidendroideae and inclusion of Spiranthoideae into Orchidoideae). In addition, whatever the classification criterion, the wide diversity in chromosome number within each subfamily of Orchidaceae added to the few cytologic data available, make it difficult to discuss the taxonomic and molecular contradictions at this level based on cytogenetic findings, although the latter proved to be a powerful tool for solving doubts regarding lower taxonomic ranks (*i.e.*, Martínez, 1985; Aoyama, 1989; Aoyama *et al.*, 1994; Felix and Guerra, 1998, 2000, 2005; Luo, 2004; this work).

Our contribution, along with the literature data, reveal that Orchidoideae (*sensu* Cameron *et al.*, 1999) is composed mainly of polyploid strains with 2*n* = 24, 26, 28, 30, 32, 36, 38, 40, 42, 44, 46, 48, 56, 64 as the most common chromosome numbers, while in Epidendroideae (*sensu* Cameron *et al.*, 1999) the polyploids 2*n* = 28, 30, 32, 34, 36, 38, 40, 42, 46, 48, 50, 52, 54, 56 are predominant, although 2*n* = 60, 84, 108 also occurr. According to Felix and Guerra (2000, 2005), x_1_ = 7 is the primary basic chromosome number for Orchidaceae, composed mostly by paleopolyploids, with the predominance of the series *n* = 7, 14, 21, 28, 42 added to dysploidy at each level. The diversity in chromosome numbers and distribution of the orchids that inhabit the northeast region of Argentina analyzed here reinforces the proposal of these authors.

Regarding chromosome size and interchromosomal asymmetry, our results point out that in Epidendroideae (*sensu* Cameron *et al.*, 1999) small chromosomes and definitely bimodal karyotypes predominate, with the exception of *Corymborkis*, of uncertain phylogenetic position, which has mostly median-size chromosomes. Furthermore, our results on Orchidoideae (*sensu* Cameron *et al.*, 1999) reveal the presence of small, median and large chromosomes, and, with the exception of *Cyclopogon* and *Stigmatosema*, distinctly bimodal karyotypes, which is more evident in *Eltroplectris*, *Mesadenella*, *Sacoila* and *Skeptrostachys*. Particularly, the chromosome size and karyotype features of the species of the Orchidoideae tribe Cranichideae [Spiranthoideae *sensu* Dressler (1993) and Szlachetko (1995)] are good markers for supporting the current lower-rank clades proposed by the latter author.

The genetic variability of the orchids is also expressed by the diversity of their chromosome number and karyotype features. Progress in the cytogenetic research of orchids is essential to solve the still persisting contradictions between the results of morphologic and molecular analyses and to help developing an improved taxonomic approach.

## Figures and Tables

**Table 1:** Chromosome numbers (2*n*) in Argentinean species of Orchidaceae.

Taxon	2n	Locality and vouchers	2*n* - Previous references
*Aspidogyne kuczyynskii* (Porsh) Garay ^2^		Ctes. Ituzaingó, Santa Tecla, Almada 151	-
*Brassavola tuberculata* Hook	40	Mnes. Capital, Posadas, Hojsgaard 228	40 - Afzelius (1943), Blumenschein (1960)
*Campylocentrum neglectum* (Rchb. f. & Warm.) Cogn.	38	Chaco, San Fernando, Colonia Benítez, Insaurralde 676	38 - Dematteis and Daviña (1999)
*Catasetum fimbriatum* (C. Morren.) Lindl. & Paxton	108	Mnes. Capital, Posadas, Insaurralde 707	108 - Jones and Darker (1968), Dematteis and Daviña (1999)
*Corymborkis flava* (Sw.) Kuntze ^1^	56	Mnes. San Pedro, P. P. Saltos del Moconá, Daviña 208	-
*Cyclopogon callophyllus* (Barb. Rodr.) Barb. Rodr.^1^	28	Mnes. Capital, Nemesio Parma, Cerutti 74	-
	28	Ctes. Ituzaingó, Garapé, Cerutti 28	-
*C. congestus* (Vell.) Hoehne	32	Mnes. Candelaria, Aº Yabebiry, Almada 150	28, 32, 36 - Martínez (1981), Tanaka and Maekawa (1983)
	32	Mnes. Aristóbulo del Valle, Cuña Pirú, Hojsgaard 192	
*C. elatus* (Sw.) Schltr.	28	Mnes. Apóstoles, Aº Chimiray, Hojsgaard 341	28, 30, 45 - Martínez (1981), Felix and Guerra (2005)
	28	Ctes. Capital, Insaurralde 708	-
*C. oliganthus* (Hoehne) Hoehne & Schltr.^1^	64	Mnes. Apóstoles, Aº Chimiray, Hojsgaard 339	-
	64	Mnes. Capital, Posadas, Aº Zaimán, Cerutti 71	
*Cyrtopodium hatschbachii* Pabst ^1^	46	Mnes. Capital, Villa Lanús, Aº Zaimán, Almada 153	-
*C. palmifrons* Rchb. f. et Warm.^1^	46	Mnes. Capital, Posadas, Hojsgaard 354	-
*Eltroplectris schlechteriana* (Porto & Brade) Pabst	26	Mnes. Montecarlo, Honfi 1357	26 - Martínez (1985), Dematteis and Daviña (1999)
*Eurystyles actinosophila* (Barb. Rodr.) Schltr.^2^	56	Mnes. Capital, Honfi 1358	-
*Galeandra beyrichii* Rchb. f.^1^	54	Mnes. Capital, Garupá, Insaurralde 705	-
*Gomesa planifolia* (Lindl.) Klotzsch ex Rchb.f.^1^	56	Mnes. San Pedro, Piñalito, Guillén 492	-
*Habenaria bractescens* Lindl.^1^	44	Mnes. Capital, Garupá, Insaurralde 697	-
*Leptotes unicolor* Bar. Rodr.	40	Mnes. San Ignacio, Teyú Cuaré, Seijo 706	40 - Blumenschein (1960)
*Mesadenella cuspidata* (Lindl.) Garay	46	Mnes. Capital, Garupá, Hojsgaard 349	46 - Martínez (1985)
	46	Ctes. Ituzaingó, Garapé, Cerutti 68	
*Miltonia flavescens* (Lindl.) Lindl.	60	Mnes. Eldorado, Valle Hermoso, Honfi 1153	60 - Sinotô (1962, 1969), Charanasri *et al.* (1973), Félix and Guerra (2000)
*Oeceoclades maculata* (Lindl.) Lindl.	56	Mnes. San Ignacio, Dematteis 486	56 - Dematteis and Daviña (1999)
	56	Mnes. Capital, Posadas, Daviña 613	48, *c.*52, 54, 58 - Guerra (1986), Felix and Guerra (2000)
*Oncidium bifolium* Sims	108	Mnes. Guaraní, Honfi 1359	108 - Dematteis and Daviña (1999)
*O. divaricatum* Lindl.^3^	56	Mnes. Bernardo de Irigoyen, Pozo Azul, Honfi 1360	42 - Charanasri *et al.* (1973)
*O. edwallii* Cogn.^1^	42	Mnes. Montecarlo, Puerto Rico, Insaurralde w/n	-
*O. fimbriatum* Lindl.^1^	56	Mnes. Bernardo de Irigoyen, Pozo Azul, Honfi 1363	-
*O. longicornu* Mutel ^3^	42	Mnes. Iguazú, Puerto Iguazú, Daviña 615	56 - Dematteis and Daviña (1999)
*O. longipes* Lindl.	56	Mnes. Iguazú, Puerto Iguazú, Daviña 616	56 - Blumenschein (1960), Dematteis and Daviña (1999)
*O. pubes* Lindl.^1^	84	Mnes. Guaraní, Cabassi w/n	-
*O. riograndense* Cogn.^1^	56	Mnes. Bernardo de Irigoyen, Pozo Azul, Honfi 1192	-
*Pelexia bonariensis* (Lindl.) Schltr.	46	Mnes. Capital, Nemesio Parma, Cerutti 29	46 - Martínez (1985), Dematteis and Daviña (1999)
	46	Mnes. Candelaria, P. P. Cañadón de Profundidad, Hojsgaard 289	
*P. ekmanii* (Kraenzl) Schltr.^1^	46	Mnes. Capital, Garupá, Radins 15	-
*P. lindmanii* Kraenzl ^1.^	46	Mnes. San Pedro, P. P. Saltos del Moconá, Daviña 123	-
*Rodriguezia decora* (Lem.) Rchb. f.	42	Mnes. Bernardo de Irigoyen, Pozo Azul, Honfi 1362	Sinoto (1962)
*Sacoila lanceolata* (Aubl.) Garay	46	Mnes. Capital, Garupá, Radins 55	46 - Cocucci (1956), Martínez (1985), Felix and Guerra (2005)
*Sarcoglottis fasciculata* (Vell.) Schltr.^3^	46, 47, 49	Mnes. Capital, Nemesio Parma, Hojsgaard 291B	46 - Martínez (1985), Felix and Guerra (2005)
	46	Mnes. Capital, Garupá, Insaurralde w/n	
	46	Ctes. Ituzaingó, Garapé, Insaurralde w/n	
*S. grandiflora* (Hook.) Klotzsch	46	Mnes. Capital, Nemesio Parma, Cerutti 56	46 - Martínez (1985)
	46	Ctes. Ituzaingó, Garapé, Insaurralde w/n	
*S. ventricosa* (Vell.) Hoehne	46	Mnes. Montecarlo, Isla Caraguatay, Hojsgaard 255	46 - Martínez (1985)
*Skeptrostachys paraguayensis* (Rchb. f.) Garay ^2^	46	Mnes. Apóstoles, San José, Baumgratz 19	-
*Sophronitis cernua* Lindl.	40	Mnes. Capital, Posadas, Honfi 1361	40 - Blumenschein (1960)
*Stigmatosema polyaden* (Vell.) Garay ^2^	40	Mnes. Apóstoles, San José, Hadad 18	-
*Trichocentrum pumilum* (Lindl.) M.W.Chase & N.H.Williams	30	Mnes. Capital, Villa Lanús, Cerutti 73	30 - Dematteis (1997), Dematteis and Daviña (1999), Felix and Guerra (2000)
*Warrea warreana* (Lodd. ex Lindl.) C. Schweinf.^1^	48	Mnes. San Pedro, P. P. Saltos del Moconá, Insaurralde w/n	-
*Zygopetalum maxillare* Lodd.	48	Mnes. Capital, Posadas, Daviña 614	48 - Blumenschein (1960), Tanaka and Kamemoto (1984)
*Zygostates alleniana* Kraenzl ^2^	54	Mnes. Apostoles, Aº Chimiray, Hojsgaard 343	-

^1^First chromosome number for the species, ^2^First chromosome number for the genus, ^3^Taxa with chromosome numbers that differ from previously published reports. Species are alphabetically grouped. All voucher specimens are deposited at MNES (Universidad Nacional de Misiones Herbarium). Mnes: Misiones, Ctes: Corrientes.
